# *In Situ* Atomic-Scale Observation of 5-Fold Twin Formation in Nanoscale Crystal under Mechanical Loading

**DOI:** 10.1021/acs.nanolett.2c03852

**Published:** 2023-01-12

**Authors:** Xiang Wang, Sixue Zheng, Chuang Deng, Christopher R. Weinberger, Guofeng Wang, Scott X. Mao

**Affiliations:** † Department of Mechanical Engineering and Materials Science, University of Pittsburgh, Pittsburgh, Pennsylvania 15261, United States; ‡ Department of Mechanical Engineering, University of Manitoba, 75A Chancellors Circle, Winnipeg, Manitoba R3T 5V6, Canada; § Department of Mechanical Engineering, Colorado State University, Fort Collins, Colorado 80524, United States

**Keywords:** 5-fold twin, *in situ* nanomechanical testing, grain boundary decomposition, partial dislocation

## Abstract

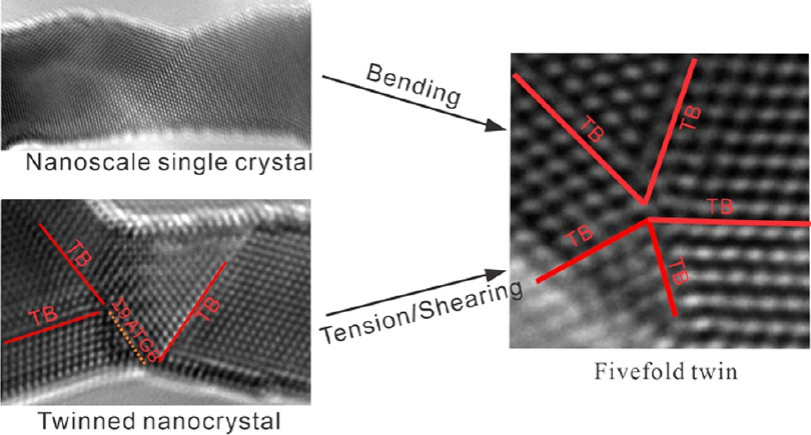

A 5-fold twin is usually observed in nanostructured metals after mechanical tests and/or annealing treatment. However, the formation mechanism of a 5-fold twin has not been fully elaborated, due to the lack of direct time-resolved atomic-scale observation. Here, by using *in situ* nanomechanical testing combined with atomistic simulations, we show that sequential twinning slip in varying slip systems and decomposition of high-energy grain boundaries account for the 5-fold twin formation in a nanoscale gold single crystal under bending as well as the reversible formation and dissolution of a 5-fold twin in a nanocrystal with a preexisting twin under tension and shearing. Moreover, we find that the complex stress state in the neck area results in the breakdown of Schmid’s law, causing 5-fold twin formation in a gold nanocrystal with a twin boundary parallel to the loading direction. These findings enrich our understanding of the formation process of high-order twin structures in nanostructured metals.

Twin boundaries (TBs) with low mobility and boundary energy can be used for tuning the mechanical properties of nanostructured metals,^1,2^ which have drawn great interest in the field of interface engineering.^3,4^ As a special twinning morphology, a 5-fold twin (FFT) with five coherent TBs concurrently meeting at their common rotation axis along [110] has been widely observed in metallic nanowires,^5−7^ nanoparticles,^8,9^ thin films,^10^ and nanocrystalline materials.^11−13^ Compared to their twin-free counterparts, the nanostructured metals with FFT usually exhibit substantially improved mechanical properties.^14−20^ It has been reported that the Young’s modulus of chemically synthesized pentatwinned silver (Ag) nanowires increased from 83 to 176 GPa^16,18^ and the yield strength increased from 0.71 to 2.64 GPa.^18^ Controlling the introduction of FFT into nanostructured metals is of vital importance for fabricating high-performance materials, which requires the knowledge of the underlying formation mechanisms of FFT.

To date, some formation mechanisms of FFT have been proposed. For instance, Liao et al.,^12,21^ An et al.^22^ and Zhu et al.^11^ reported that high external stress, an orientation change in applied stress (ball milling and high-pressure torsion), and low experimental temperature were required to activate partial dislocation activities from grain boundaries (GBs) and TBs on different twinning systems, causing FFT formation in nanocrystalline metal materials. Furthermore, Huang et al.,^23^ Cao et al.,^24^ and Bringa et al.^25^ demonstrated that FFT formed with zero external stress in nanocrystalline metals at high temperature during annealing treatment, where the splitting and migration of a GB segment,^23,26^ grain rotation,^24^ stacking fault (SF) motion and overlapping,^24^ and successive partial emission driven by high internal stresses at TBs and GBs^25^ were the dominant mechanisms. However, the formation mechanisms of FFT proposed in these previous studies were all deduced from the post-mortem observation, which fell short in obtaining direct time-resolved atomic-scale observation. Complementary to the experimental observation, molecular dynamics (MD) simulations^27,28^ demonstrated that the size nonuniformity and the different orientations of the constituent grains in nanocrystalline metals induced a complex stress state in the grain interior under uniaxial tension, facilitating the operation of different twinning systems and the consequent formation of FFT. Given that the FFTs observed in prior studies were often related to the complex GB structures of the deformed nanocrystalline metals and the complex stress states under extreme loading modes,^11,12,25,27^ we postulate whether FFT could be formed in a single crystal under simple loading modes, such as uniaxial tension and bending. In addition, all the previous studies focused on investigating the mechanisms of FFT formation. The dynamic process of FFT dissolution and their atomic-scale mechanisms remains unexplored, due to the significant experimental challenge.

Here, we use a gold (Au) nanocrystal as a model system to investigate the atomic-scale formation process of FFT by conducting *in situ* nanomechanical tests and imaging using high-resolution transmission electron microscopy (HRTEM) combined with atomistic simulations. The results demonstrate that sequential twinning on several slip systems and the decomposition of grain boundaries (GBs) at the node of a multifold twin play a critical role in FFT formation in a Au single crystal under bending and the reversible formation and dissolution of FFT in a Au nanocrystal with a preexisting twin under tension and shearing. Moreover, the complex stress state in the neck area results in FFT formation in a gold nanocrystal with a twin boundary parallel to the loading direction, different from the prediction of Schmid’s law. Our findings provide deep insights into FFT formation under mechanical loading, which are of importance for microstructure control to improve the mechanical properties of metal materials.

Figure 1 and Movie S1 show the atomic-scale process of FFT formation in a single-crystalline Au nanocrystal during bending. A nanoscale Au single crystal is prepared inside a TEM instrument by nanowelding (see the Supporting Information). As shown in Figure 1a, the nanocrystal is loaded along the [111] direction with a displacement rate of ∼10^–2^ nm/s and viewed along the [1̅10] zone axis, which allows direct observation of perfect and partial dislocations. Prior to the *in situ* bending test, the as-fabricated Au nanocrystal has negligible strain (Figure S1a,c). Upon bending, the maximum tensile and compressive strains in the nanocrystal before yielding are up to ∼4% (Figure S1b,d), which are comparable to the elastic strain limit of 2.1–5.3% for a Au nanocrystal.^29^ Further bending deformation gives rise to surface nucleation and glide of partial dislocations on the (111) and (1̅1̅1) slip planes, leaving behind stacking faults (SFs), as marked with the red arrows in Figure 1b,c and Figure S2a,b. Some of the partial dislocations are found to meet at the center of the nanocrystal, where the lengthwise stress is zero. The nucleation of additional partial dislocations, primarily on (1̅1̅1), leads to the formation of a low-angle GB^30^ (the dashed line in Figure 1c). With further bending, partial dislocation activities on the other slip planes occur (Figure 1d,e and Figure S2c). The GB angle also increases from 12 to 16°, a direct result of the lattice reorientation caused by a partial dislocation slip.^24^ As the bending deformation increases, additional partial dislocations nucleate between the SFs, producing a 4-fold twin (the red lines in Figures 1f–h and Figure S2d)^12^ and a 20° GB (Figure 1h). Subsequently, the GB reorganizes itself into a coherent TB by absorbing and emitting partial dislocations, and the TBs migrate, reducing the high elastic energy associated with the incompatibility of the separated TBs in the newly formed FFT (Figure 1i and Figure S2e–g).^22^ Distinct from the experimental observation of FFT formation under bending, the formation of a single deformation twin and the occurrence of a perfect dislocation slip were often observed in the single-crystalline Au and Ag nanowires under ⟨110⟩ uniaxial tensile loading.^31−33^ Such different twinning behavior is attributed to the complex stress state induced by bending, which favors the operation of different twinning systems.

**Figure 1:**
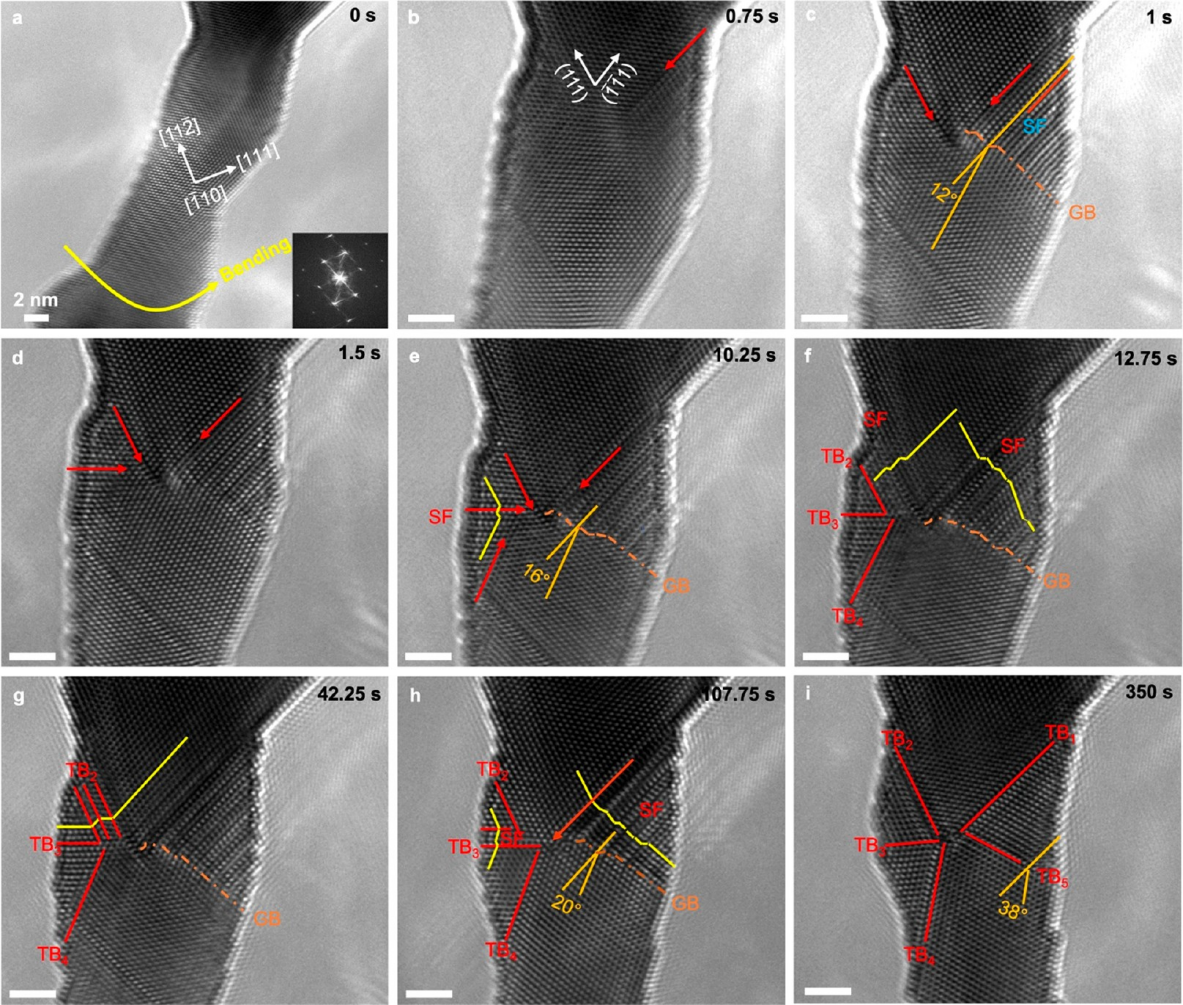
Bending-induced 5-fold twin formation in a single-crystalline Au nanocrystal. (a) TEM image of a single-crystalline Au nanocrystal. (b–e) Successive emission of partial dislocations on four different twinning systems in the Au nanocrystal. The GB angle increases from 12 to 16° upon bending. (f) Transformation of SF bundles into the twin variants of the 3-fold twin. (g) TB migration mediated by partial dislocation slip. (h) Formation of a 4-fold twin. (i) Formation of a 5-fold twin through lattice reorientation. All of the scale bars are 2 nm.

To understand the atomic-scale formation process of FFT under bending, MD simulations of nanoscale Au single crystal were performed (Figure 2 and Movie S2). The applied bending force on the nanocrystal is along the [110] direction (CD' in Figure S2f,g). The bending deformation induces compressive and tensile stress at the two opposite sides of the Au nanocrystal, respectively (Figure S3a). Given that the leading partial has a higher Schmid factor when the ⟨001⟩-oriented nanocrystal is under compression rather than tension, a partial dislocation slip is favored in the compressive side of the nanocrystal.^34,35^ Under a compressive stress of ∼8.6 GPa, plenty of partial dislocations are observed to nucleate from the compressive side of the free surface on (11̅1̅) planes, glide in the nanocrystal, and then terminate around the neutral plane with zero lengthwise stress (Figure S3b), resulting in the formation of a TB (T1 in Figure 2b and ABD in Figure S2f,g) and a GB (Figure 2a,b). Meanwhile, a severely distorted area, comprised of the atomic columns colored in gray and red in Figure 2b, forms at the tensile side of the nanocrystal to accommodate the imposed bending deformation. With increasing strain, a part of the high-energy distorted area under the tensile stress of ∼5.9 GPa (Figure S3b,c) relaxes into a twin with TB on the (1̅1̅1) plane (T3 in Figure 2c and ABD′ in Figure S2g) through atom readjustment, lowering the overall system energy.^36^ The unrelaxed region of the disordered area is indicated by the gray and red atomic columns in Figure 2c. Subsequently, the GB evolves into a TB on the (111) plane (T2 in Figure 2d and ABC in Figure S2g), due to lattice reorientation mediated by partial dislocation emission and absorption at the GB with a local stress as high as ∼8.6 GPa (Figure S3b–d). Accompanied with the formation of a 3-fold twin, a Σ27 GB appears at the node of the newly formed 3-fold twin (Figure 2e), and subsequently it decomposes into two TBs during further deformation^8^ (T4 and T5 in Figure 2f and Figure S3e,f), leading to FFT formation (Figure S2g).

**Figure 2:**
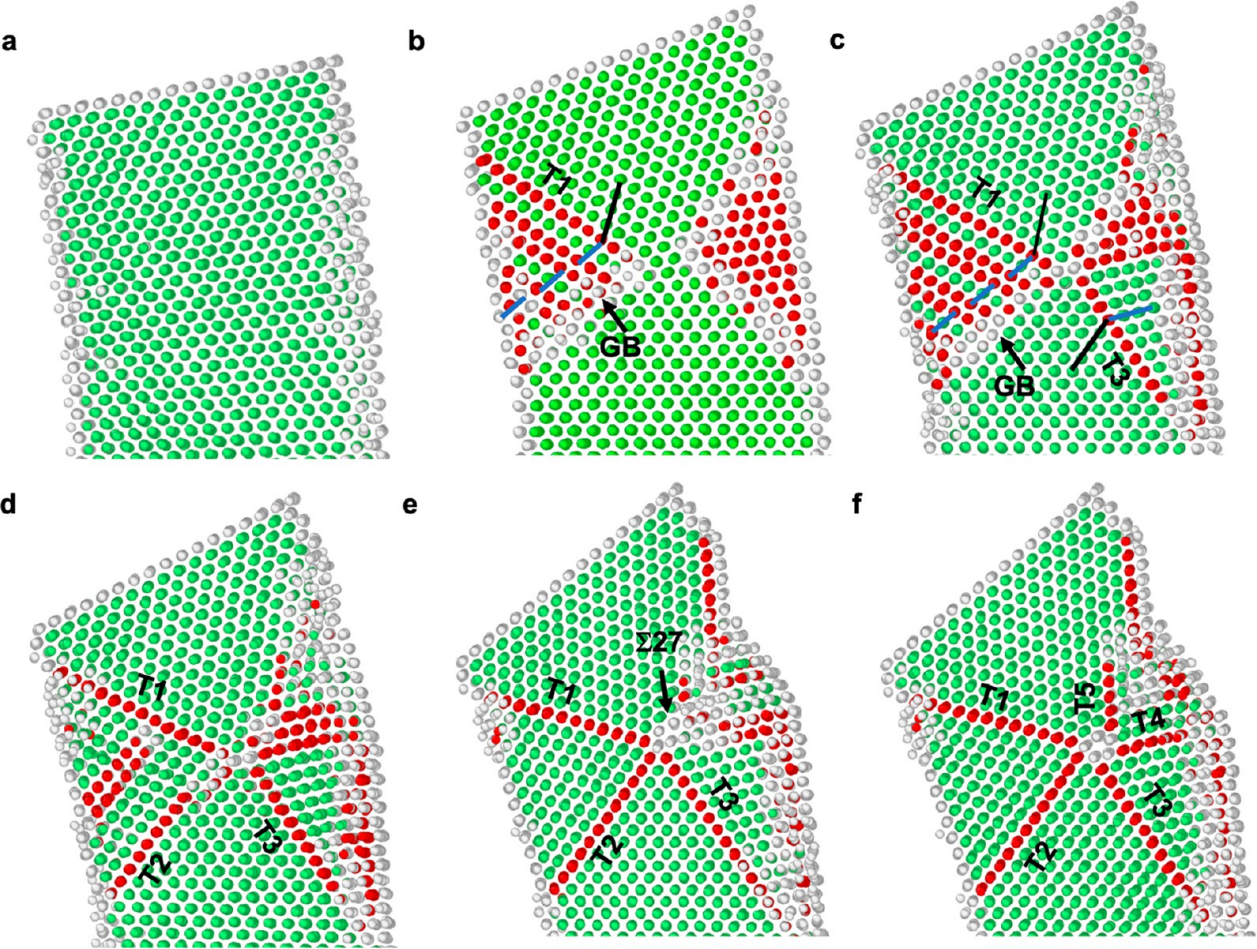
MD simulation showing the atomic-scale process of 5-fold twin formation in a single-crystalline Au nanocrystal upon bending. (a) The bending Au nanocrystal before defect nucleation. (b) Successive partial dislocations emit from the free surface and terminate near the neutral plane, resulting in the formation of a TB and a GB. (c) Transformation of a distorted area into a twin via atom adjustment. (d) Formation of a 3-fold twin through partial dislocation slip-mediated lattice reorientation. (e) Formation of a Σ27 GB at the node of the 3-fold twin. (f) Formation of two TBs caused by the decomposition of Σ27 GB. Based on a common neighbor analysis,^50^ the atoms with face-centered cubic (FCC), hexagonal-close-packed (HCP), and other structures are colored in green, red, and gray, respectively.

The different pathways for FFT formation in the experimental and computational results are probably related to the surface conditions of the Au nanocrystals. The nanoscale Au single crystal in the experiment is fabricated via *in situ* nanowelding (see the Supporting Information), probably introducing some mass-deficient defects at the surface of the “pristine” nanocrystal, which are preferential dislocation nucleation sites.^37,38^ Moreover, the diameter of the as-fabricated Au nanocrystal is not as uniform as that in the MD simulations. Numerous atomic-scale steps exist at the free surface of the Au nanocrystal. Upon mechanical loading, stress concentration could appear at the surface step, facilitating a dislocation slip.^39,40^ In contrast, the surface of the Au nanocrystal in MD simulations is atomically flat. Hence, a partial dislocation slip is prone to be activated during *in situ* bending tests, compared to that in MD simulations. Despite the different surface conditions and orders of magnitude difference in loading rate and time scale between MD simulations and experiments, both the experimental and computational results show that a 5-fold twin could form in the nanoscale Au single crystal under bending, which is energetically favorable.

Not in the single-crystalline Au nanocrystal under uniaxial tension,^31,32,40^ but in the Au nanocrystal with a preexisting twin under shearing and tension, FFT is observed to form (Figure 3 and Movie S3). As shown in Figure 3a, a Au nanocrystal with a 4-fold twin is subjected to shear loading along the [111] direction under a strain rate of 10^–3^ s^–1^. Upon shear loading, partial dislocations sequentially glide along the preexisting SF and are absorbed into the TB (TB_4_ in Figure 3a), resulting in the nucleation and growth of a twin in domain III and thus generation of a FFT (Figure 3a,b). The absorption of partial dislocations into TB_4_ results in the emission of partial dislocations from TB_4_ (Figure 3a) into domain IV, leading to the migration of TB_6_ and the consequent extension of domain IV. When the shearing direction is reversed, detwinning occurs in domains III and IV through the successive slip of partial dislocations along the TBs (TB_3_ and TB_6_ in Figure 3c–g) in the opposite direction. Consequently, four TBs, i.e., TB_3_, TB_4_, TB_5_ and TB_6_, annihilate, producing an Σ9 asymmetric tilt GB that extends from the node of the FFT to the nanocrystal surface. The FFT is transformed into a triple junction of a V-shaped twin and a Σ9 GB (Figure 3c–g). The formation and dissolution of FFT can be controlled by applying and removing shear, or equivalently reversing the shear direction. Moreover, the as-formed triple junction can be transformed back into a FFT under tensile loading along the [110] direction at a strain rate of 10^–3^ s^–1^ (Figure 3h–j). On the basis of the change in (110) interplanar spacing during mechanical loading, the applied tensile stress is estimated to be 1.7 GPa (see the Supporting Information). Considering that the largest Schmid factor for leading partial dislocation in Au nanocrystal under ⟨110⟩ tension is 0.47, the resolved shear stress on the ⟨112⟩{111} slip system is 0.8 GPa, which is comparable to the shear stress of 0.47 GPa required for partial dislocation nucleation in a Au nanowire.^39^ Partial dislocation slip could be activated in the Au nanocrystal upon tensile deformation. As shown in Figure 3i, the surface nucleation and glide of a partial dislocation generates a SF in the Au nanocrystal. Subsequently, the consecutive slip of twinning partials nearby the SF results in twin formation in domain IV (Figure 3j). Concurrently, the Σ9 GB decomposes into two TBs^41^ (TB_3_ and TB_4_ in Figure 3j), transforming the V-shaped twin-triple junction back into a FFT. During the whole process of FFT formation and dissolution, the morphology of the V-shaped twin, composed of TB_1_ and TB_2_, remains unchanged, indicating that there is no interaction between dislocations and these two TBs. The twin morphology of the newly formed FFT could be further altered via partial dislocation slip along TBs and dislocation transmission through TB under shear deformation (Figure 3k–l).^42^ Hence, the formation, dissolution, and even morphology of FFT could be controlled through partial dislocation slip along TBs and the decomposition and generation of GBs. To demonstrate the universality of the FFT formation mechanism described above, an *in situ* tensile test of a Au nanocrystal with a preexisting 2-fold twin was performed along the [112] direction at room temperature under a strain rate of 10^–3^ s^–1^ (Figure S5). Under tensile loading, the decomposition of Σ9 GB and a series of twinning dislocation activities at the intersection of the 2-fold twin result in FFT formation.

**Figure 3:**
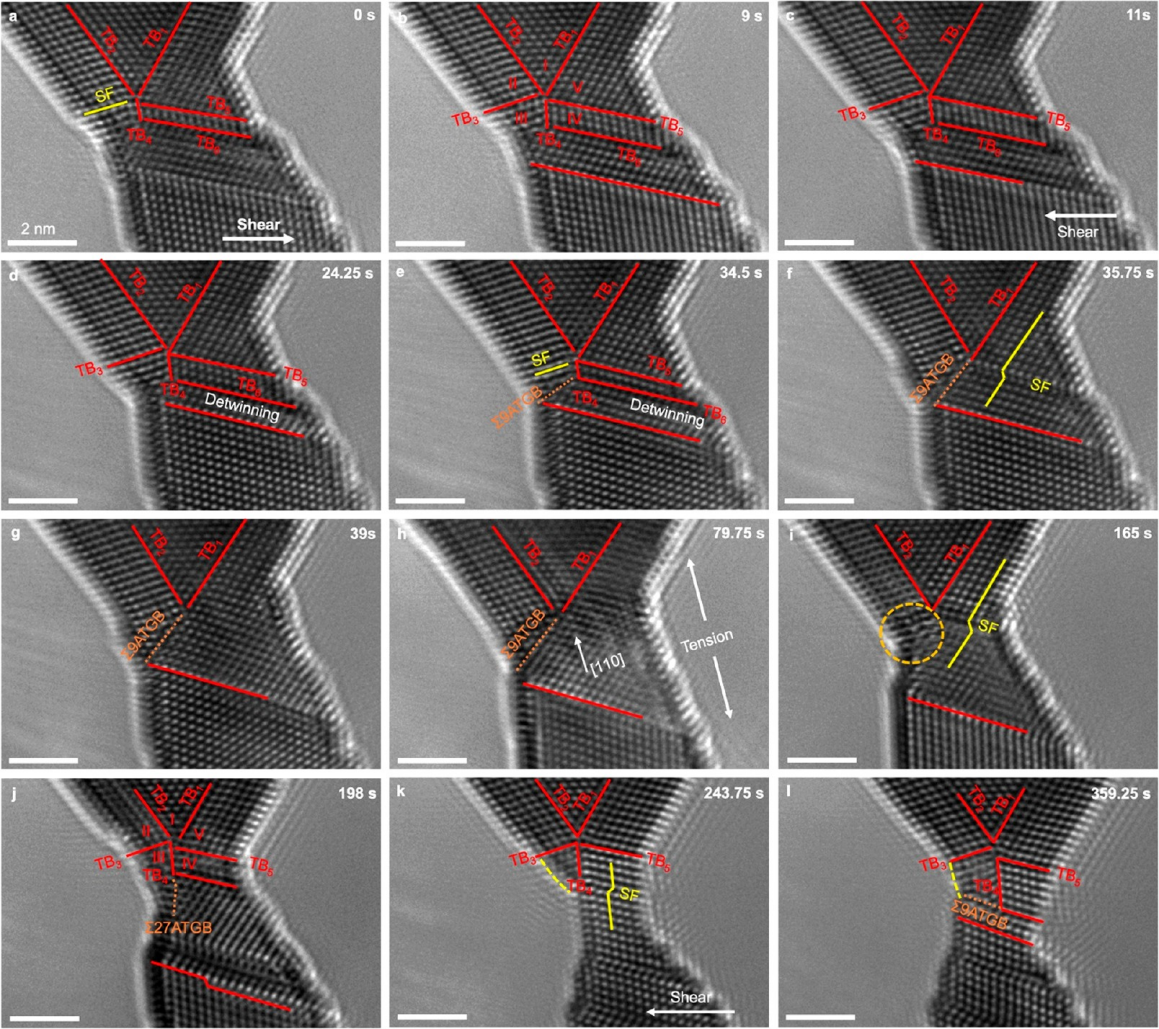
Shear- and tension-induced formation and dissolution of a 5-fold twin in a multifold twinned nanocrystal. (a) An as-fabricated Au nanocrystal with a 4-fold twin structure. The nanocrystal is viewed along [1̅10]. (b) Formation of a 5-fold twin via successive twinning partial slip along the preexisting SF and TBs. (c–g) Detwinning of a 5-fold twin upon reverse shear loading. A 2-fold twin and a Σ9 GB form after the detwinning process. (h–j) Formation of a 5-fold twin through partial dislocation slipping and Σ9 GB decomposition under tensile loading at a strain rate of ^1^0^–3^ s^–1^. (k, l) Change in twin morphology of the 5-fold twin upon shear loading. All of the scale bars are 2 nm.

To further understand the critical role of GB decomposition in FFT formation, atomistic simulations of a Au nanocrystal with a triple junction of a ∑9 {001}/{112} asymmetric tilt GB and a TB were conducted (Movie S4). When the Au nanocrystal is compressed along the [001] direction, the segments of Σ9 GB above and below the TB decompose into four TBs (Figure 4a,b). As the deformation proceeds, partial dislocation emission at Σ9 GBs and free surfaces are observed, resulting in the migration of the newly formed TBs and the consequent formation of FFT (Figure 4c,d). With further deformation, the as-formed FFT rearranges itself through a partial dislocation slip, reducing the overall system energy (Figure 4d–f). A similar process of FFT formation, mediated by GB decomposition and partial dislocation slip, is also found in a Au nanocrystal with a Σ9 {111}/{115} asymmetrical tilt GB during compression (Figure S6). Moreover, our MD simulations show that there are two possible ways for Σ9 GB to decompose. In the Σ9 {111}/{115} asymmetrical tilt GB, the two decomposed TBs are parallel and inclined to the initial Σ9 GB (Figures 4g,h), while in the Σ9 {001}/{112} asymmetrical tilt GB, the two decomposed TBs are both inclined to the initial Σ9 GB (Figures 4i,j).

**Figure 4:**
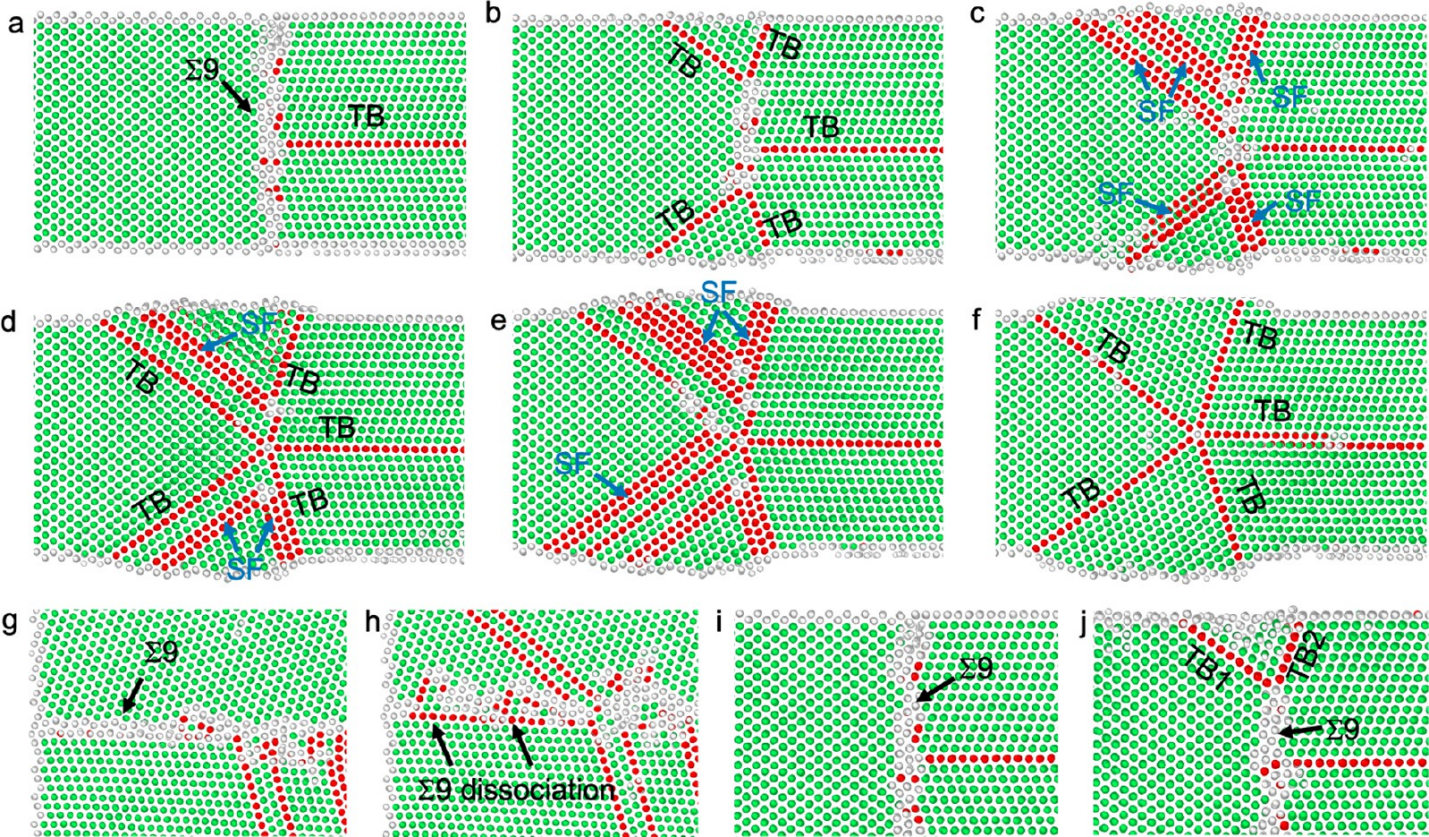
MD simulation showing atomic-scale processes of 5-fold twin formation in the Au nanocrystal and Σ9 GB decomposition. (a) A Au nanocrystal with a TB intersected with a Σ9 GB under ⟨112⟩ compression. (b) Formation of four TBs via Σ9 GB decomposition upon compression. (c–f) Adjustment of the FFT via a series of partial dislocation slip from the free surface and Σ9 GBs. (g–j) Atomic scale processes of the decomposition of Σ9 {111}/{115} (g, h) and Σ9 {001}/{112} (i ,j) asymmetrical tilt GBs. Based on a common neighbor analysis,^50^ the atoms with FCC, HCP, and other structures are colored in green, red, and gray, respectively.

Figure 5a,b shows the atomically resolved process of FFT formation in the Au nanocrystal with a TB parallel [112̅] loading direction under a strain rate of 10^–3^ s^–1^. A neck exists in the bitwinned Au nanocrystal, as shown in Figure 5a. After tensile failure, a FFT is observed to form in the fractured Au nanocrystal (Figure 5b), which is reproducible in a different but similarly bitwinned Au nanocrystal (Figure S7). The formation of this kind of FFT took place so rapidly that its evolution was not recorded in our experiments. Thus, to further understand the formation mechanisms of the FFT, we used atomistic simulations of a bitwinned Au nanocrystal to model this process (Figure 5c–f). The bitwinned nanowires are constructed along the ⟨112⟩ direction and deformed at room temperature and a strain rate of 10^8^ s^–1^ (Movie S5). Upon tensile loading, a perfect dislocation slip on the inclined (11̅1) and (1̅11) planes dominates the plastic deformation (Figure 5c), which can be well explained by a quantitative analysis of the Schmid factors for the perfect and partial dislocation slip on {111} slip planes. The Schmid factors are 0.408 for perfect dislocation slip ([011̅](11̅1̅) and [101̅](1̅11̅)) larger than those of 0.393 for partial dislocation slip ([121̅] (11̅1̅) and [211̅] (1̅11̅)). Upon initiation of plastic deformation, a large number of perfect dislocations nucleate in a localized region, resulting a neck in the nanocrystal (Figure 5d). During further loading, a partial dislocation slip on the (1̅1̅1) plane is observed, resulting in the nucleation and growth of deformation twins and the ultimate formation of a FFT (Figure 5e,f). Notably, the initial twinning elements of the formed FFT in the bitwinned Au nanocrystal are the [112̅](111) type, the activation of which leads to a contraction rather than an elongation of the nanocrystal. Moreover, the Schmid factor for a 1/6[1̅1̅2̅] partial dislocation slip on the (1̅1̅1) plane in the [112̅]-oriented nanocrystal under tension is −0.314. Hence, perfect and partial dislocation slip on other inclined {111} slip planes are favored over a partial dislocation slip that would lead to FFT formation. This indicates that the formation of FFT in the bitwinned Au nanocrystal is specific to the deformation in the neck region. No similar behavior is observed in our experiments or simulations outside of this neck region. Consequently, it is concluded that the complex stress state in the neck and the need for material reorientation in this region favor the FFT formation in the neck area just prior to tensile failure.

**Figure 5:**
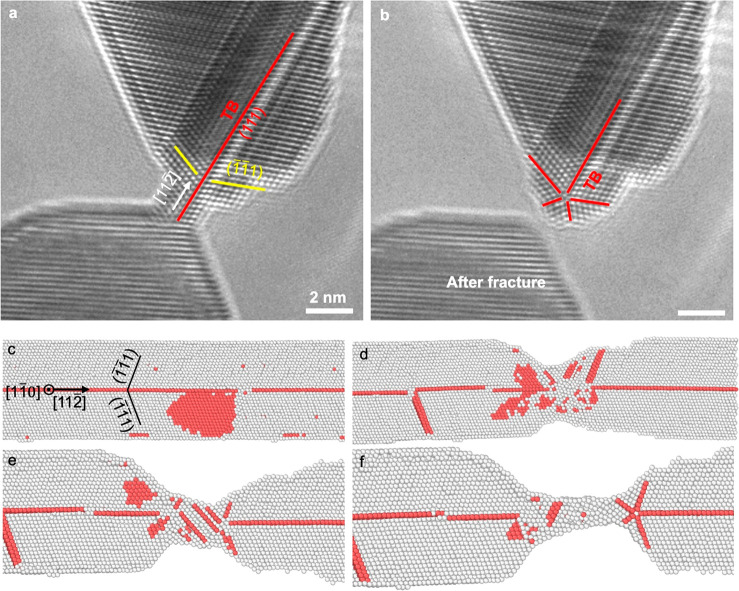
FFT formation in the neck area of a Au nanocrystal with a TB parallel to the tensile loading direction. (a, b) *In situ* atomic-scale observation of FFT formation in the neck of the Au nanocrystal loaded along ⟨112⟩ direction at room temperature under a strain rate of 10^–3^ s^–1^. The nanocrystal is viewed along [1̅10]. All of the scale bars are 2 nm. (c) MD snapshot showing dislocation activities on inclined {111} planes. Based on a common neighbor analysis,^50^ the atoms with an HCP structure are colored in red, and the atoms with other structures are colored in gray. (d) Formation of a neck in the bitwinned Au nanocrystal at the final stage of the tensile test. (e, f) Series of MD snapshots showing FFT formation in the neck area of a Au nanocrystal.

Distinct from FFT formation in nanocrystalline metals under extreme loading conditions (ball milling and high-pressure torsion),^11,12,21^ our work reveals that FFT could be formed in a single-crystalline Au under bending, which is attributed to the following reasons. First, localized stress concentration exists near surface steps in the nanoscale Au single crystal upon mechanical loading, favoring surface nucleation and glide of partial dislocations on the ⟨112⟩{111} slip system.^39^ The emission of partial dislocations from the preferential surface sites on non-neighboring {111} planes results in the nucleation and growth of deformation twins unfollowing the traditional layer-by-layer fashion.^43,44^ Different from the *in situ* bending tests, the high strain rate employed in the atomistic simulations accounts for the untraditional twinning route during the process of FFT formation. Second, a bending deformation induces lattice rotation and complex stress state in the sample (Figure S1d and Figure S3a), which favors the activation of partial dislocation activities in varying slip systems,^11−13,21^ causing FFT formation in a single-crystalline metal. For the Au nanocrystals with preexisting defects (twins and GBs) under uniaxial tension or simple shearing, the different orientations of the constituent crystal lattice induced a complex stress state, facilitating the operation of different twinning systems and thus causing the formation of FFT.

In addition to the partial dislocation slip, GB decomposition also plays a critical role in FFT formation. Partial dislocation activities in different slip systems usually cause the formation of 2-fold and 3-fold twins in nanostructured metals.^3,45^ Geometrically necessary GBs, that is Σ9 with a GB energy of ∼542 mJ/m^2^^45^ and Σ27 with an energy of ∼560 mJ/m^2^,^45^ are subsequently generated at the node of two- and three-order twins, respectively, which lead to a substantial increase in excessive elastic strain energy. From the point of view of thermodynamics, the high-energy Σ9 and Σ27 GBs are prone to decompose into two Σ3 GBs (coherent TB) with relatively low energy (∼17.5 mJ/m^2^ ^46^), reducing the overall system energy.^45^ A 3-fold twin could directly develop into a FFT through Σ27 GB decomposition, while partial dislocation slipping is needed to be activated to cooperate with Σ9 GB decomposition causing the transition from a 2-fold twin to a FFT. The experimental observations of transformation from 2- and 2-order twins to a 5-fold twin in this study are energetically favorable.^47^

Our findings reveal the pathways for FFT formation in Au nanocrystals with different microstructural features. Given that introducing FFT into nanostructured metals greatly improved their mechanical properties,^14−16^ deep insights into the atomic-scale formation processes of FFT are of vital importance for providing new concepts to design high-performance metal materials. Considering that previous studies mostly focused on the mechanical behavior of the chemically synthesized metallic nanowire with the common ⟨110⟩ axis of a 5-fold twin parallel to the loading direction,^6,17,18,48,49^ the approaches for mechanically introducing FFT into the nanoscale Au, reported in this study, open up new avenues to investigate the intrinsic deformation features of FFT.

In conclusion, combined *in situ* TEM experiments and atomistic simulations reveal that partial dislocation activities in varying slip systems and the decomposition of a high-energy grain boundary are responsible for the formation of a 5-fold twin in a nanoscale Au single crystal under bending and the reversible formation and dissolution of a 5-fold twin in Au nanocrystals with a preexisting twin under shearing and tension. Moreover, the complex stress state in the neck leads to 5-fold twin formation in a bitwinned Au nanocrystal, disobeying Schmid’s law. Our work provides atomistic insights into the formation process of a 5-fold twin in a single-crystal metal under mechanical loading, which paves the way for rationally manipulating microstructure features to fabricate high-performance nanostructured metals.

## Data Availability

The data used in this study are available from the corresponding author upon request.

## Supporting Information Available

Strain analysis of the Au nanocrystal during bending, schematics showing 5-fold twin formation in a single-crystalline Au nanocrystal under bending, quantitative stress mappings of the nanoscale Au single crystal under bending in MD simulations, tensile stress of a Au nanocrystal under tensile deformation at a strain rate of 10^–3^ s^–1^, 5-fold twin formation in a Au nanocrystal with a 2-fold twin under ⟨112⟩ tensile loading at room temperature at a strain rate of 10^–3^ s^–1^, MD simulation showing 5-fold twin formation in a Au bicrystal, 5-fold twin formation in the Au nanotip after the tensile failure of the bitwinned Au nanocrystal, measurement of lattice stress in Au nanocrystal during tensile tests, and materials and methods (PDF)
Bending-induced 5-fold twin in a nanoscale Au single crystal (AVI)
Molecular dynamics simulation of 5-fold twin formation in single-crystalline Au nanocrystal under bending (MOV)
Shear/tension-induced 5-fold twin in a multifold-twinned nanocrystal (AVI)
Molecular dynamics simulation of decomposition of Σ9 grain boundary (MOV)
Molecular dynamics simulation of 5-fold twin formation in a bitwinned Au nanocrystal under tensile loading (MOV)

## Supplementary Material

nl2c03852_si_001.pdf

nl2c03852_si_002.avi

nl2c03852_si_003.mov

nl2c03852_si_004.avi

nl2c03852_si_005.mov

nl2c03852_si_006.mov
