# Supplementary material for: In Situ Atomic-Scale Observation of 5-Fold Twin Formation in Nanoscale Crystal under Mechanical Loading
Source: Nano Lett. 2023 Jan 12;23(2):514–22. doi: 10.1021/acs.nanolett.2c03852 (PMC10032584; doi:10.1021/acs.nanolett.2c03852)
Supplement: Supplementary file 1 — nl2c03852_si_001.pdf [file nl2c03852_si_001.pdf]

Supplementary Information for
In situ atomic-scale observation of fivefold twin formation in nanoscale crystal under mechanical loading

Xiang Wang[1†], Sixue Zheng[1†], Chuang Deng[2*], Christopher R. Weinberger[3], Guofeng Wang[1*] and Scott X. Mao[1*]

[1]Department of Mechanical Engineering and Materials Science, University of Pittsburgh, Pittsburgh, Pennsylvania 15261, USA

[2]Department of Mechanical Engineering, University of Manitoba, 75A Chancellors Circle, Winnipeg, MB R3T 5V6, Canada

[3]Department of Mechanical Engineering, Colorado State University, Fort Collins, CO, 80524, USA

[†]These authors contributed equally: Xiang Wang, Sixue Zheng.

*Corresponding authors. E-mail: chuang.deng@umanitoba.ca (C.W.), guw8@pitt.edu (G.W.), sxm2@pitt.edu (S.X.M);

Supplementary Information includes:

1. Figures S1-7

2. Measurement of lattice stress in Au nanocrystal during tensile test

3. Supplementary information for Materials and Methods

4. Captions for Movies S1-5

5. Supplementary References

1. Supplementary Figures

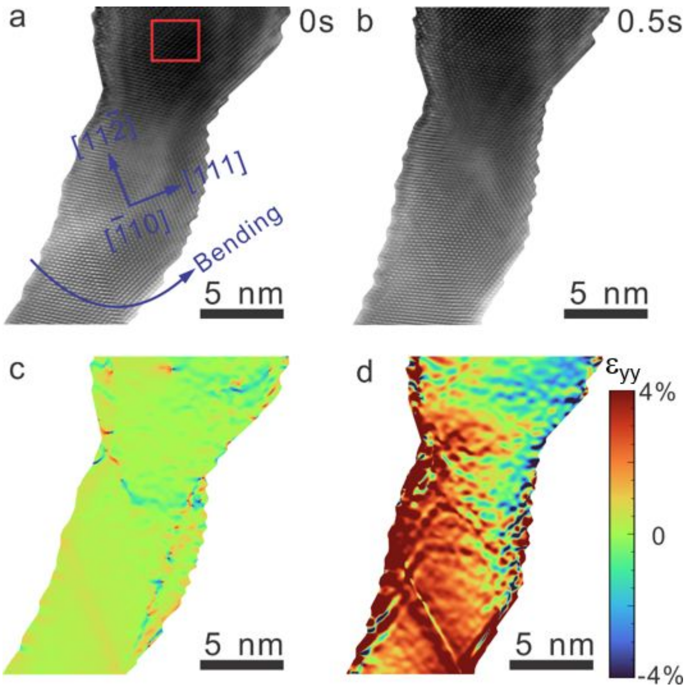

Figure S1. Strain analysis on the Au nanocrystal during bending. (a-b) TEM images of the Au nanocrystal before (a) and after (b) bending deformation. (c-d) Quantitative strain mappings of the Au nanocrystal before (c) and after (d) bending deformation, by geometrical phase analysis. The unstrained lattice in the red framed region in (a) serves as a reference for the geometrical phase analysis (see ref.[1] for more details). The color variation from dark blue to dark red indicates the variation of stain values from -4% to 4%.

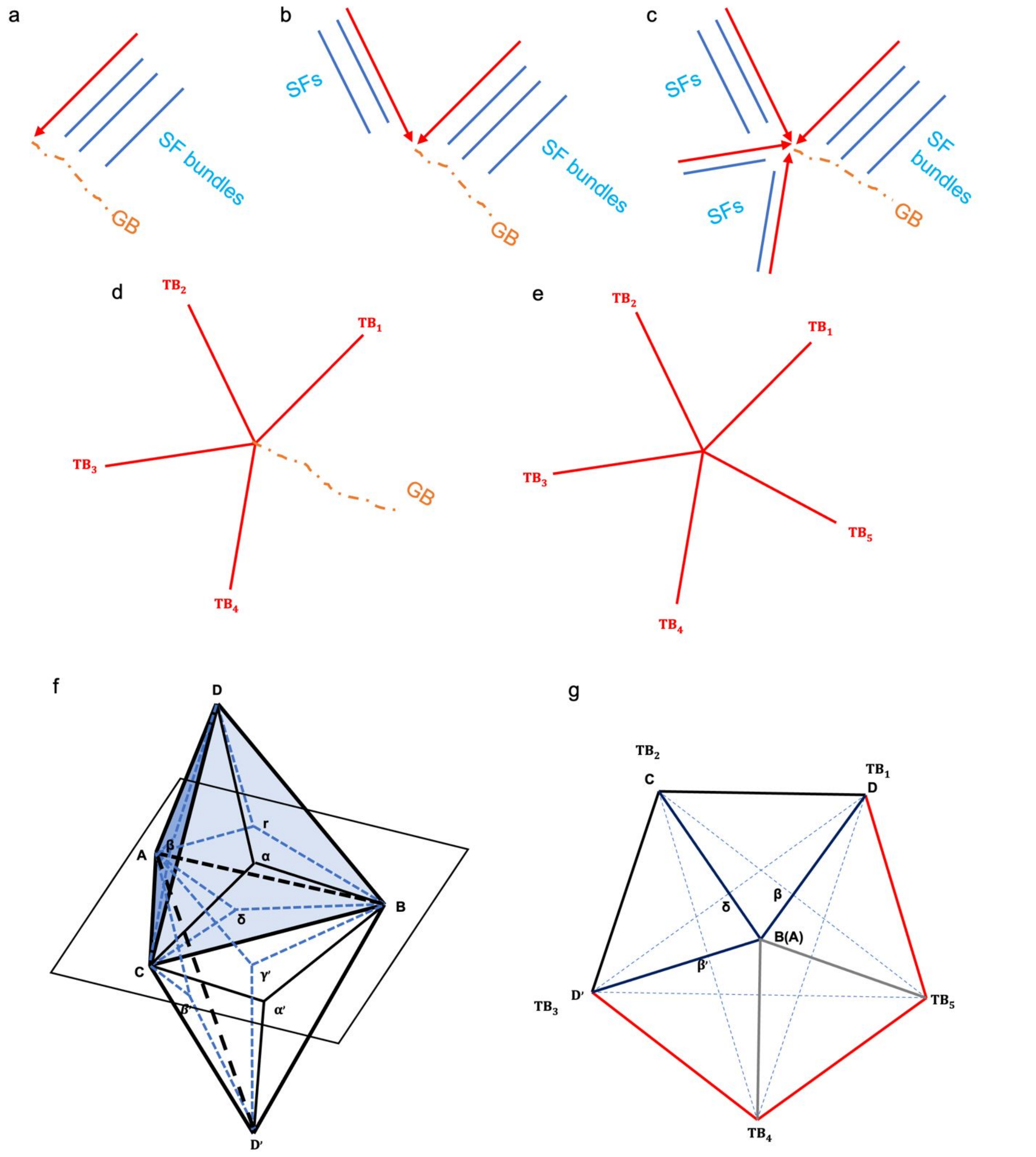

Figure S2. Schematics showing fivefold twin formation in a single crystalline Au nanocrystal under bending. (a-c) Successive emission of partial dislocations on four different twinning systems in the Au nanocrystal upon bending resulting in the formation of stacking fault bundles.

(d-e) Transformation from GB to TB in a fourfold twin resulting in the formation of a fivefold twin. (f-g) Thompson tetrahedrons used for illustrating fivefold twin.

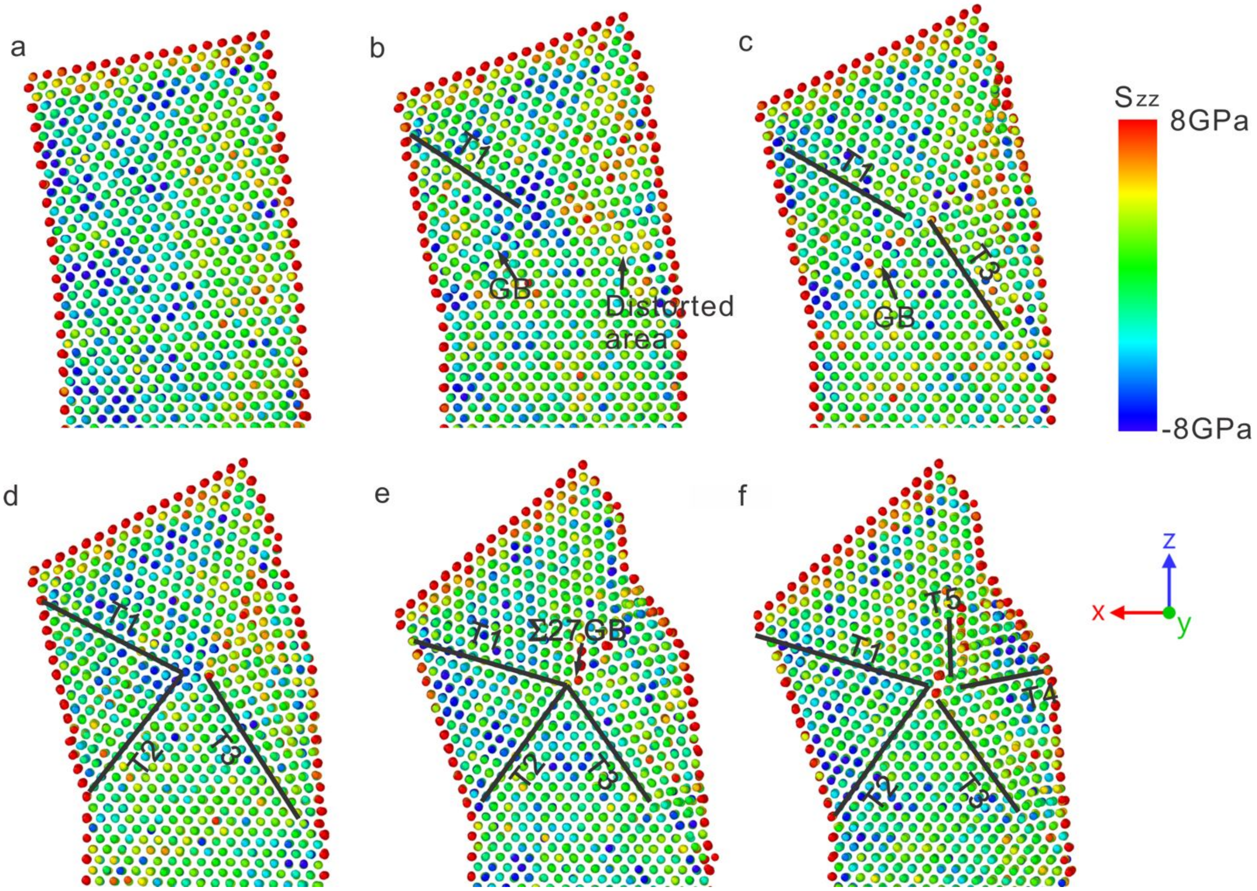

Figure S3. Quantitative stress mappings of the nanoscale Au single crystal under bending in MD simulation. (a) Au nanocrystal before defect nucleation. The high compressive stress of ~ 8.6 GPa appears near free surface. (b) Formation of a TB and a GB caused by partial dislocation slip. Meanwhile, a distorted area forms in the area under the tensile stress of ~4.9 GPa. (c) Transformation of the distorted area into a twin via atom adjustment. (d) Formation of threefold twin by absorbing and emitting partial dislocations at GB under the compressive stress of ~6.6 GPa. (e) Formation of a Σ27 GB at the node of the threefold twin. (f) Formation of two TBs caused by the decomposition of Σ27 GB under the tensile stress of ~9.8 GPa. The color variation from blue to red corresponds to the stress from -8 GPa to 8 GPa.

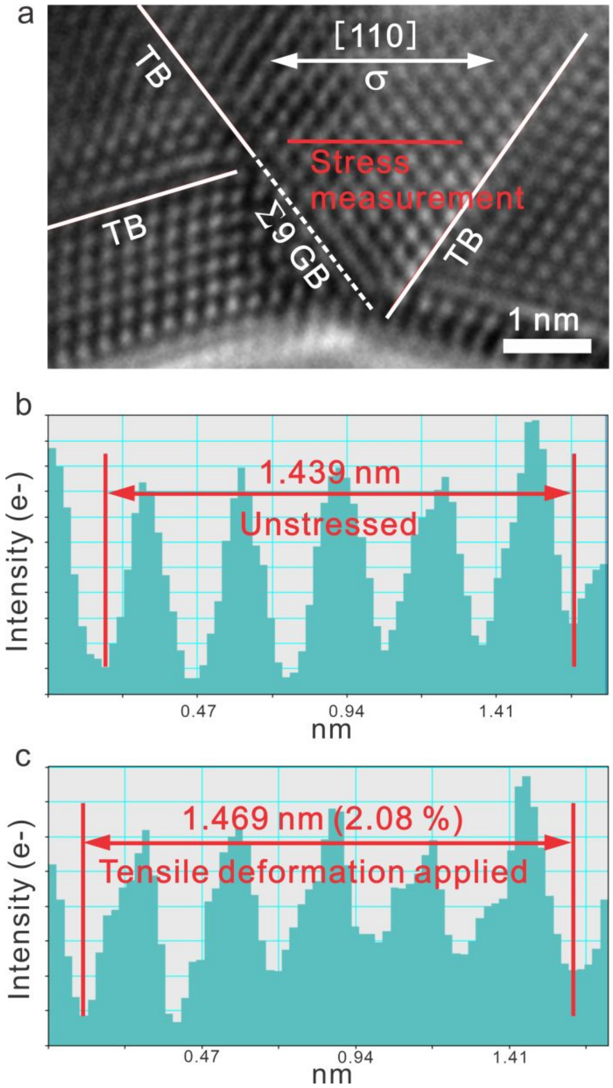

Figure S4. Tensile stress of Au nanocrystal under tensile deformation at a strain rate of 10^{-3} s^{-1}. (a) Au nanocrystal is under [110] tensile loading. (b-c) The intensity profile of the atomic columns along loading direction. The 5-planes measurements of the lattice spacing along [110] loading are (b) 1.439 nm in the unstressed nanocrystal and (c) 1.469 nm in the deformed nanocrystal. Lattice strain of 2.08% is obtained by measuring the change in the (110) interplanar spacing.

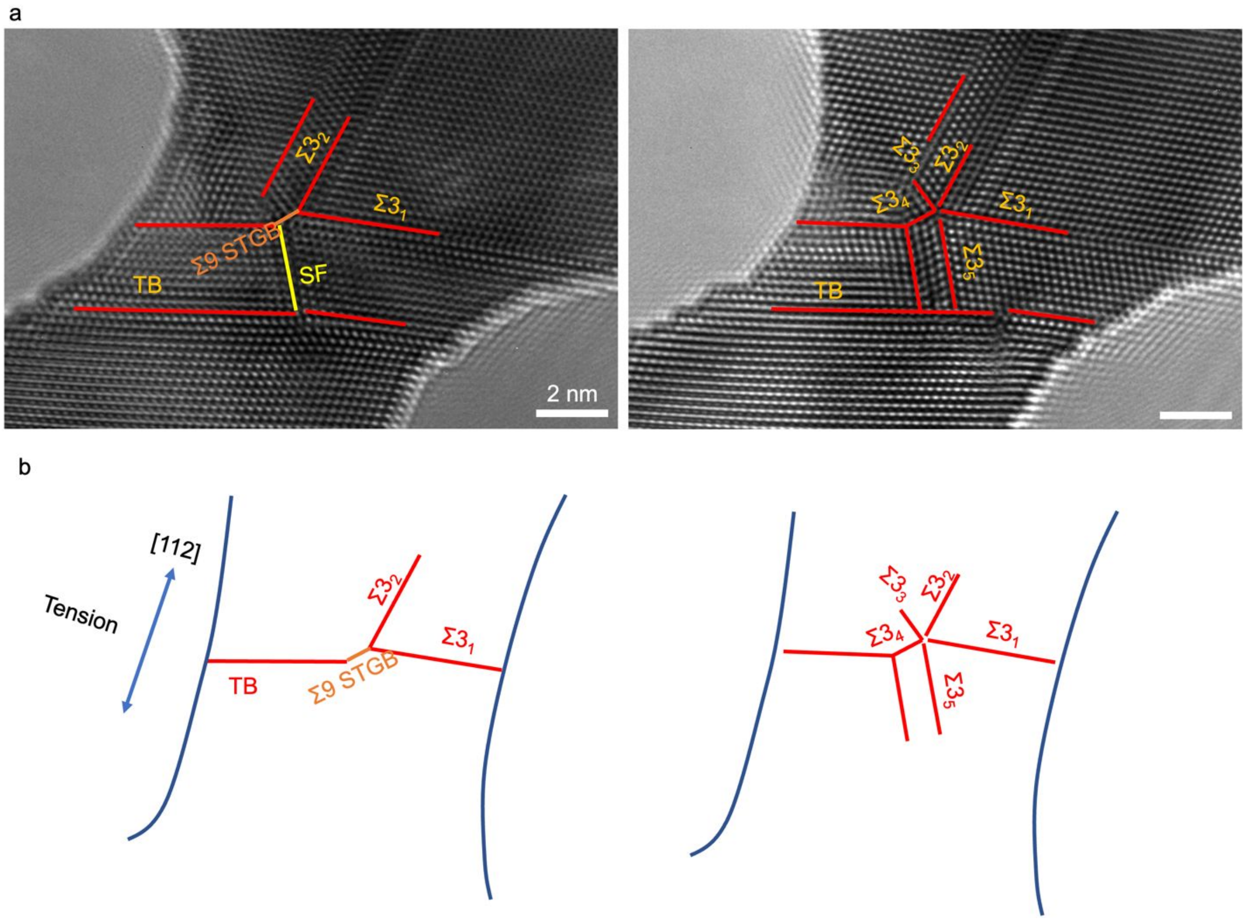

Figure S5. Fivefold twin formation in a Au nanocrystal with a twofold twin under <112> tensile loading at room temperature under a strain rate of 10^{-3} s^{-1}. (a) The decomposition of Σ9 GB and partial dislocation slipping resulting in the formation of a FFT. All the scale bars are 2 nm. (b) Schematics showing the formation of fivefold twin resulting from Σ9 GB decomposition and partial dislocation slipping.

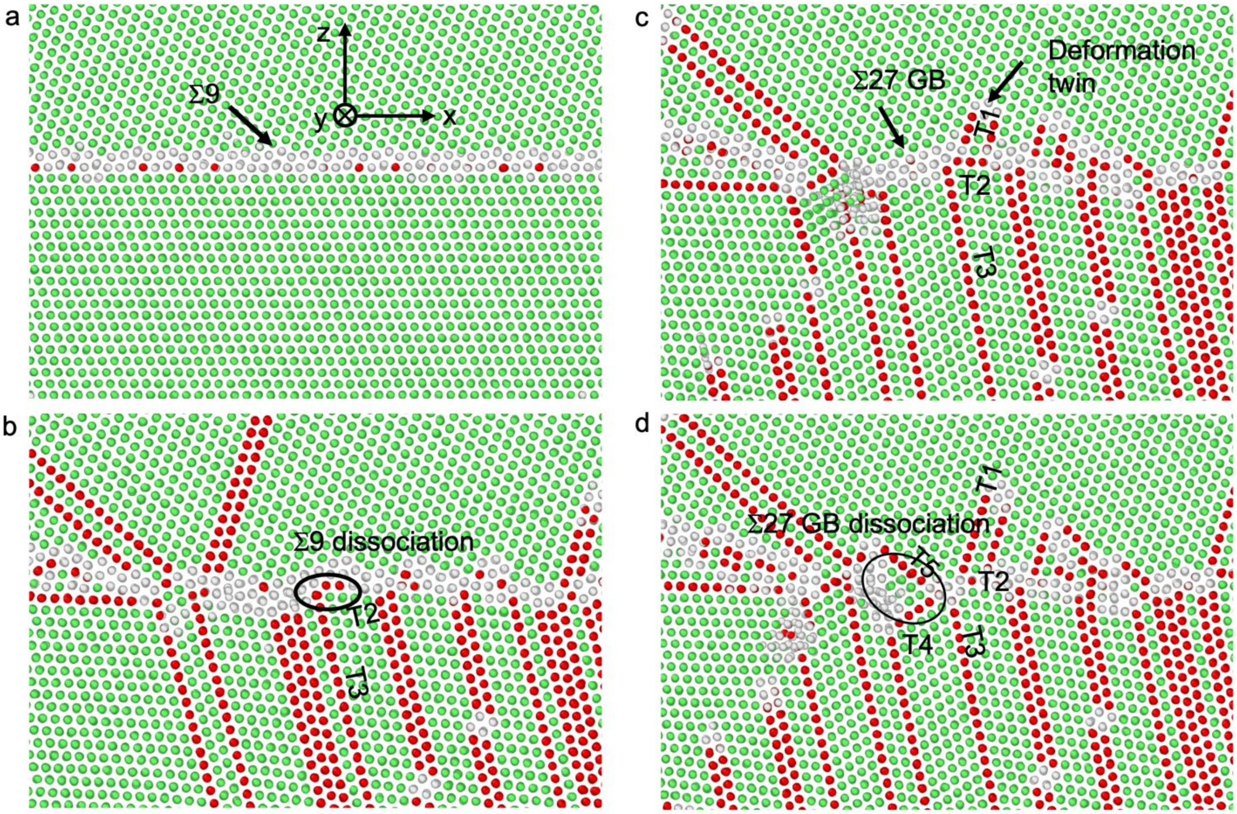

Figure S6. MD simulation showing fivefold twin formation in a Au bicrystal. (a) A pristine Au nanocrystal with a Σ9 {111}/{115} asymmetrical tilt GB. (b) Formation of two TBs through Σ9 GB decomposition. (c) Partial dislocation activities at GB causing the formation of a threefold twin and a Σ27 GB. (f) Σ27 GB decomposition at the node of the threefold twin, resulting in the formation of fivefold twin in the Au nanocrystal upon compression. The five TBs are indicated as T1, T2, T3, T4 and T5, respectively.

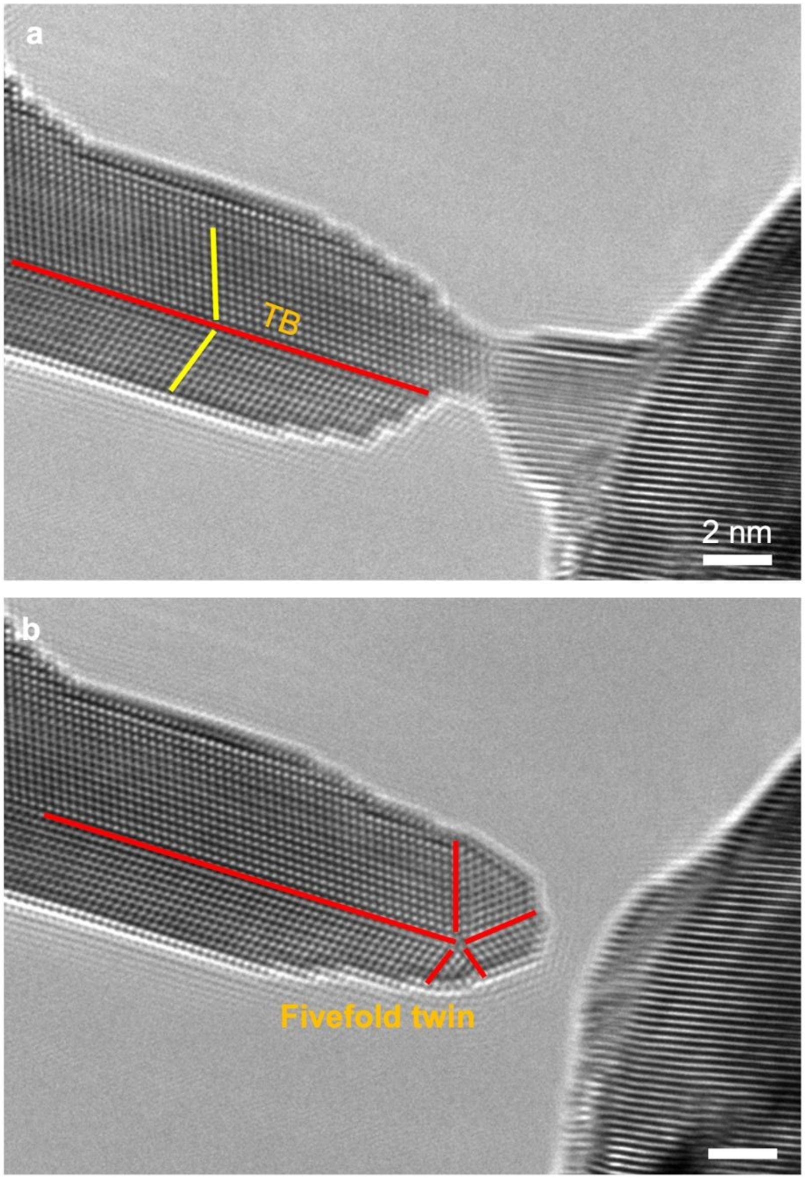

Figure S7. Fivefold twin formation in the Au nanotip after the tensile failure of the bi-twinned Au nanocrystal. All the scale bars are 2 nm.

2. Measurement of lattice stress in Au nanocrystal during tensile test

The tensile stress is measured at the atomic scale on the basis of the measured lattice strain and the Young's modulus along [110]-direction (Figure S4a)[2,3]. To analyze the lattice strain during tension, the lattice spacings of the unstressed and deformed states along [110]-loading direction are determined in a TEM software of DigitalMicrograph® developed by Gatan Inc (Figure S4b-c). The tensile lattice strain of 2.08% is obtained by comparing the change in (110) interplanar spacing during tension, as shown in Figure S4b-c. Besides, the Young's modulus (E_{ijk}) for a [ijk]-oriented single crystal with cubic structure is given by[4]

$$\frac{1}{E_{\mathrm{ijk}}} = S_{11} - 2\left(S_{11} - S_{12} - \frac{1}{2}S_{44}\right) \times \left(l_{i1}^2 l_{j2}^2 + l_{j2}^2 l_{k3}^2 + l_{i1}^2 l_{k3}^2\right), \tag{S1}$$

where S_{11}, S_{12} and S_{44} are elastic compliances and l_{i1}, l_{j2} and l_{k3} are the direction cosines of the loading direction [ijk]. Considering that the elastic compliances for Au single crystal at room temperature are S_{11}=2.33×10^{-2} GPa^{-1}, S_{12}=-1.065×10^{-2} GPa^{-1} and S_{44}=2.38×10^{-2} GPa^{-1} [4], the Young's modulus for [110]-oriented Au is calculated to be 81.5 GPa. Thus, the tensile stress is determined to be 1.7 GPa by multiplying the Youngs' modulus of [110]-oriented Au nanocrystal (81.5 GPa) with the measured lattice strain (2.08%).

3. Supplementary information for Materials and Methods

In situ TEM nanomechanical testing

All the *in situ* mechanical tests of Au nanocrystals were performed inside a FEI Titan TEM using a Nanofactory scanning tunneling microscope (STM) holder. The Au rod investigated here were provided by ESPI Metals, which have a high purity of 99.999%. Before the mechanical tests, Au nanotips were generated at the fracture surface of a Au rod using a wire cutter. Subsequently, the

fractured Au rod with numerous nanotips and a W nanoprobe were loaded onto the TEM holder, serving as the static and probe sides, respectively. The sharp Au nanotips oriented in the <110> zone axis were selected to be contacted with the W probe via controlling the movement of the piezo-manipulator. The Au nanotip and W probe were then welded together via applying a constant voltage of ~1 V, generating bridge-shaped nanowires with controllable dimensions and microstructure features. The strain rate applied during mechanical loading was controlled by the movement speed of the piezo-manipulator of the STM holder. All the *in situ* experiments were recorded in real-time by a charge-coupled device (CCD) camera at a rate of 0.25 s/frame.

MD simulations

All the computational simulations were performed by using Large-scale Atomic/Molecular Massively Parallel Simulator (LAMMPS) package[5] with an embedded-atom method potential for Au[6]. This potential has been well documented to produce consistent simulation results with experimental observations in Au nanowires under various loading conditions[7]. Prior to the mechanical deformation, the energy of the models was minimized by the conjugate gradient method, and then relaxed at 300 K for 25 ps under a canonical (NVT) ensemble. Atomic structures of the Au nanocrystals during mechanical loading were visualized, using common neighbor analysis (CNA) for the defect structure in OVITO[8]. The atomic stress in the Au nanocrystal can be analyzed by using the Virial theorem as discussed by Cheung and Yip[9].

In total, four models have been constructed for atomistic modeling and simulation by using Atomsk[10]. The first model (Model 1 as shown in Figure 2) is a <112>-oriented square Au nanowire with the width and length being ~ 5 and 16 nm, respectively. The second model (Model 2 as shown in Supplementary Figure 5) is a bicrystalline model in Au containing an asymmetric <110>-tilt Σ9

{111}/{115} GB with the dimensions being ~ 30, 2.9, and 10 nm along the x, y, and z directions, respectively. The third model (Model 3 as shown in Figure 5) is a square Au nanowire with dimensions being ~ 11 and 20 nm along the width and length, respectively, which contains a triple junction in the middle. The GB between the two grains on the right side is a coherent twin boundary, while the two GBs between the left grain and the two grains on the right are both asymmetric <110>-tilt {001}/{112} GB with the misorientation angle being 35.26°, which is close to the 38.94° for an ideal Σ9 GB. The Model 1 was subject to bending test, which was achieved by rotating a thin slab with thickness ~ 1nm at both the top (clockwise) and bottom (anti-clockwise) of the nanocrystal around its respective center of mass along the <110> direction (i.e., the y-axis) at a constant speed of 0.05°/ps. For Models 2 and 3, compression was applied along the x-axis by moving a thin slab with thickness ~1 nm at both the top and bottom at a constant speed of 1 m/s towards each other. Periodic boundary condition was applied along the <110>-tilt direction (i.e., the y-axis) in Model 2 during both the relaxation and mechanical loading, while free boundary conditions were applied for all other cases. The time step was 5 femto-second and the Nose/Hoover thermostat was used to control the temperature[11, 12].

The fourth model is a bi-twinned Au nanocrystal with axes along a <112> direction constructed by applying the twin symmetry across a {111} plane that lies along the axis of the nanocrystal (Figures 3c-f). The diameter of the nanocrystal is around 4 nm, similar in size to those observed in experiments. In order to mitigate surface and or facet effects, the cross-section of the were chosen to be circular. However, both variable diameter and other cross-sections were also tested. For constant cross-section nanocrystal, periodic boundary conditions were applied along the length of the nanocrystal, while the lateral surfaces of the wire were exposed to vacuum. The nanocrystals were then run for 1 million timesteps, with a time step of 1 femto-second, at a

temperature of 300K using a Nove'-Hoover thermostat. The stress along the axis of the nanowire was controlled to be zero using a Parinello-Rahman barostat. After equilibration, the nanocrystals were then deformed using a constant strain rate simulation (non-equilibrium molecular dynamics) by integrating the equations of motion with the temperature controlled by a Nose-Hoover thermostat.

4. Captions for supplementary movies

Movie S1. Bending-induced fivefold twin in a nanoscale Au single crystal.

Movie S2. Molecular dynamics simulation of fivefold twin formation in single-crystalline Au nanocrystal under bending.

Movie S3. Shear/Tension-induced fivefold twin in a multifold-twinned nanocrystal.

Movie S4. Molecular dynamics simulation of decomposition of Σ9 grain boundary.

Movie S5. Molecular dynamics simulation of fivefold twin formation in a bi-twinned Au nanocrystal under tensile loading.

5. Supplementary References

1. Hÿtch, M.; Snoeck, E.; Kilaas, R., Quantitative measurement of displacement and strain fields from HREM micrographs. *Ultramicroscopy* **1998,** *74* (3), 131-146.
2. Zheng, S.; Shinzato, S.; Ogata, S.; Mao, S., Experimental molecular dynamics for individual atomic-scale plastic events in nanoscale crystals. *Journal of the Mechanics and Physics of Solids* **2022,** *158*, 104687.
3. Wang, J.; Zeng, Z.; Weinberger, C. R.; Zhang, Z.; Zhu, T.; Mao, S. X., In situ atomic-scale observation of twinning-dominated deformation in nanoscale body-centred cubic tungsten. *Nature Materials* **2015,** *14* (6), 594-600.
4. Meyers, M. A.; Chawla, K. K., *Mechanical behavior of materials*. Cambridge university press: 2008.
5. Thompson, A. P.; Aktulga, H. M.; Berger, R.; Bolintineanu, D. S.; Brown, W. M.; Crozier, P. S.; in't Veld, P. J.; Kohlmeyer, A.; Moore, S. G.; Nguyen, T. D., LAMMPS-a flexible simulation tool for particle-based materials modeling at the atomic, meso, and continuum scales. *Computer Physics Communications* **2022,** *271*, 108171.

6. Grochola, G.; Russo, S. P.; Snook, I. K., On fitting a gold embedded atom method potential using the force matching method. *The Journal of chemical physics* **2005,** *123* (20), 204719.
7. Wang, J.; Sansoz, F.; Huang, J.; Liu, Y.; Sun, S.; Zhang, Z.; Mao, S. X., Near-ideal theoretical strength in gold nanowires containing angstrom scale twins. *Nature Communications* **2013,** *4* (1), 1-8.
8. Stukowski, A., Visualization and analysis of atomistic simulation data with OVITO–the Open Visualization Tool. *Modelling and simulation in materials science and engineering* **2009,** *18* (1), 015012.
9. Cheung, K. S.; Yip, S., Atomic-level stress in an inhomogeneous system. *J Appl Phys* **1991,** *70* (10), 5688-5690.
10. Hirel, P., Atomsk: A tool for manipulating and converting atomic data files. *Computer Physics Communications* **2015,** *197*, 212-219.
11. Nosé, S., A unified formulation of the constant temperature molecular dynamics methods. *The Journal of chemical physics* **1984,** *81* (1), 511-519.
12. Hoover, W. G., Canonical dynamics: Equilibrium phase-space distributions. *Physical review A* **1985,** *31* (3), 1695.
